# A Novel Small Molecule, LCG-N25, Inhibits Oral Streptococcal Biofilm

**DOI:** 10.3389/fmicb.2021.654692

**Published:** 2021-03-29

**Authors:** Xiaoying Lyu, Chungen Li, Jin Zhang, Liang Wang, Qingsong Jiang, Yusen Shui, Lan Chen, Youfu Luo, Xin Xu

**Affiliations:** ^1^State Key Laboratory of Oral Diseases, National Clinical Research Center for Oral Diseases, West China Hospital of Stomatology, Sichuan University, Chengdu, China; ^2^State Key Laboratory of Biotherapy and Cancer Center, West China Medical School, West China Hospital, Sichuan University, Chengdu, China; ^3^Department of Cariology and Endodontics, West China Hospital of Stomatology, Sichuan University, Chengdu, China

**Keywords:** dental caries, oral biofilm, *Streptococcus mutans*, antimicrobial small molecule, cytotoxicity

## Abstract

Dental caries is a chronic oral infectious disease caused by cariogenic biofilm adhered on the tooth surface. Our previous study demonstrated that a repurposed natural compound napabucasin (NAP) showed good antimicrobial activity against oral streptococcal biofilms. The current study designed a novel small molecule, namely LCG-N25, using NAP as a lead compound, and aimed to investigate its potential as an antimicrobial agent in the control of dental caries. LCG-N25 was designed and synthesized with reference to the structure of NAP. The minimal inhibitory concentrations and the minimal bactericidal concentrations of LCG-N25 against *Streptococcus mutans*, *Streptococcus sanguinis*, and *Streptococcus gordonii* were evaluated by microdilution method. The antimicrobial activity of LCG-N25 was further evaluated by crystal violet staining, colony forming units counting, biofilm metabolism assay, dead/live fluorescent staining, and scanning electron microscopy. The effect of LCG-N25 on the extracellular polysaccharides of biofilms was determined by both anthrone-sulfuric acid method and fluorescent staining. The microbial composition of streptococcal biofilms after LCG-N25 treatment was further visualized and quantified by fluorescence *in situ* hybridization. Besides, the cytotoxicity of LCG-N25 was evaluated by Cell Counting Kit-8 assay, and repeated exposure of *S. mutans* to LCG-N25 treatment was performed to assess if this novel compound could induce drug resistance of this cariogenic bacterium. We found that LCG-N25 exhibited a good antibacterial activity, low-cytotoxicity, and did not induce drug resistance of cariogenic *S. mutans*. These findings suggest that LCG-N25 may represent a promising antimicrobial agent that can be used as an adjuvant to the management of dental caries.

## Introduction

Dental caries is one of the most common chronic infectious diseases that occur at any age of humans (Gao et al., 2018). *Streptococcus mutans* is well-recognized as the main causative factor of dental caries due to its acidogenicity and aciduricity, as well as its ability to synthesize exopolysaccharides (EPS) that mediate microbial adhesion on the tooth surface (Marsh, 2010). Recent studies have also indicated the involvement of oral commensals, such as *Streptococcus sanguinis* and *Streptococcus gordonii*, in the development of dental caries (Kreth et al., 2008). Dental plaque biofilm is a three-dimensional accumulation of oral microbes and extracellular matrix adhered to the enamel surface (Hall-Stoodley and Stoodley, 2009; Karatan and Watnick, 2009). Unlike planktonic cells, microbial biofilms display increased tolerance to the host defenses and antimicrobial agents (Marsh, 2010; Algburi et al., 2017), challenging the clinical management of dental caries. The commonly used method for caries prevention was mechanical plaque control, such as tooth brushing and flossing. However, it relies heavily on personal compliance. Topical use of antimicrobials is considered as an appropriate combinatory measure for the control of dental caries, particularly in the high-risk population (Rath and Singh, 2013). Chlorhexidine (CHX) is one of the most common antimicrobial agents. It is recognized as the principal agent for chemical plaque control (Jones, 1997). However, CHX has cytotoxic effects on a wide variety of human cells including oral mucosal cells, blood cells, keratinocytes, osteoblasts, and osteoclasts (Ribeiro et al., 2004; Cabral and Fernandes, 2007). Besides, CHX can cause taste confusions, tooth staining, and drug resistance. Thereby, alternative antibacterial agents are still needed for the control of dental plaque and caries.

Small molecules have shown inhibitory effect on bacterial biofilms due to its good antimicrobial activity, good stability, and low toxicity (Worthington et al., 2012). In our previous study, we screened from a library of bioactive molecules against *S. mutans* and identified a natural compound napabucasin (NAP) (2-acetylfuro-1,4-naphathoquinone) isolated from *Newbouldia laevis*, showing antimicrobial activity against oral streptococci (Kuang et al., 2020). However, NAP as an antitumor treatment currently in phase III clinical trials shows mild cytotoxic effects on oral cells, and its antimicrobial activities against oral streptococci are relatively lower than CHX. Hence, we redesigned and synthesized a novel molecule with NAP as a lead compound, namely LCG-N25, which is expected to exhibit stronger antimicrobial activity and lower cytotoxicity. The current study aimed to examine the antimicrobial activity of this novel compound against oral streptococci. The cytotoxicity and possible drug resistance of this compound were further evaluated. We found that LCG-N25 exhibited potent antimicrobial activity against oral streptococci in both planktonic culture and biofilms, and showed low cytotoxicity against human oral cells. In addition, repeated treatment with LCG-N25 did not induce drug resistance in *S. mutans*.

## Materials and Methods

### Synthesis of LCG-N25

**FIGURE 1:**
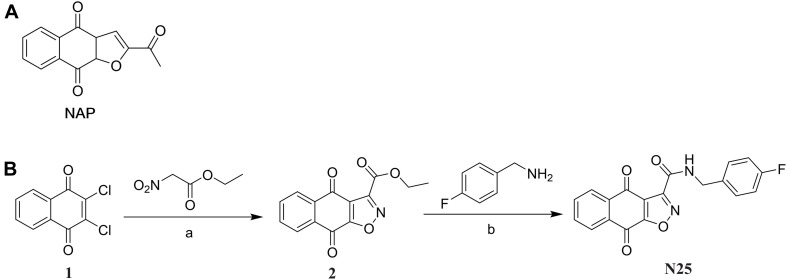
**(A)** Chemical structures of compound NAP. **(B)** Synthesis of compound LCG-N25. Reagents and conditions: **(a)** DIPEA, N2, EtOH, reflux; **(b)** MeOH, reflux.

As shown in Figure 1, the synthesis of the target compound LCG-N25 was accomplished in two steps:

#### Synthesis of Intermediate 2

The intermediate 2 was prepared according to the previously reported procedure (Santos et al., 2010). To a solution of 2,3-dichloronaphthalene-1,4-dione (500 mg, 2.2 mmol, 1.0 equiv), diisopropylethylamine (851 mg, 6.6 mmol, 3.0 equiv) in anhydrous ethanol, ethyl nitroacetate (878 mg, 6.6 mmol, 3.0 equiv) was added. The reaction solution was refluxed under N_2_ atmosphere protection for 6 h, and then concentrated under reduced pressure. The residue was purified by chromatography on Si gel column with PE/EA to give intermediate 2 as a red solid (80 mg, 29%) (Supplementary Figure 1).

#### Synthesis of the Target Compound LCG-N25

To a solution of intermediate 2 (271 mg, 1 mmol, 1.0 eq) in MeOH (4 mL), (4-fluorophenyl) methanamine (156 mg, 1.25 mmol, 1.25 eq) was added. The mixture was stirred at 64°C for 3 h. Then the solution was concentrated under reduced pressure, and the residue was purified by chromatography on Si gel column with DCM/MeOH to afford LCG-N25 as a white solid (168 mg, 48%) (Supplementary Figures 2–4).

### Tested Bacterial Strains and Chemicals

*Streptococcus mutans* UA159, *Streptococcus gordonii* DL1, and *Streptococcus sanguinis* ATCC 10556 were kindly provided by the State Key Laboratory of Oral Diseases (Sichuan University, Chengdu, China). The bacteria were grown in Brain Heart Infusion Broth (BHI, Oxoid, Basingstoke, United Kingdom) at 37°C under 5% CO_2_ (v/v). When needed, the medium was supplemented with 1% (*w/v*) sucrose (designated BHIS broth). LCG-N25 was dissolved in DMSO at a stocking concentration of 100 mg/mL.

### Bacteria Inoculation and Biofilm Formation

The planktonic bacteria were recovered from −80°C and incubated overnight at 37°C, and then diluted to 1 × 10^5^ colony forming units (CFU)/mL in BHI. BHI supplemented with 1% sucrose (BHIS) was used for biofilm formation. For multi-species biofilm formation, *S. mutans*, *S. gordonii*, and *S. sanguinis* were mixed at a ratio of 1:1:1 (*v/v/v*) in BHIS. In all the assays based on 96-well cell culture plates, the LCG-N25 solution’s volume added to each well was 100 μL and the bacterial dilution added to each well was 100 μL. In all the assays based on 24-well cell culture plates, the LCG-N25 solution’s volume added to each well was 0.5 mL, which was the same as the bacterial dilution added to each well. In all the assays based on 12-well cell culture plates, the volume of LCG-N25 solution added to each well was 1 mL, while the same volume of bacterial dilution was added to each well. In all the assays based on the chemotaxis chamber μ-Slide, the volume of LCG-N25 solution added to each well was 125 μL, while the same volume of bacterial dilution was added to each well.

### Assessment of Minimal Inhibitory Concentrations and Minimal Bactericidal Concentrations

Minimal inhibitory concentrations (MICs) and minimal bactericidal concentrations (MBCs) were examined by the microdilution method, as described previously (Nudera et al., 2007; Xu et al., 2011). Briefly, LCG-N25 was two-fold diluted with BHI in 96-well plates, the overnight culture of *S. mutans, S. sanguinis*, and *S. gordonii* was diluted to 1 × 10^5^ CFU/mL with BHI and then added to the plates. The final concentrations of LCG-N25 ranged from 0.0625 to 128 μg/mL. The final concentration of DMSO was set at 0.128% in each experimental and control group. 200 μL of BHI containing equal concentration of DMSO and bacterial suspension was used as a negative control. CHX was used as a positive control. The plates were incubated for 24 h at 37°C to assess LCG-N25′s inhibitory effect. The OD600  nm value of each well was evaluated by a microplate reader (Power Wave 200 Microplate Scanning Spectrophotometer; Bio-TeK Instruments Inc., Winooski, VT, United States) and the Windows-based computer program KC4 Data Analysis Software (Bio-TeK Instruments, Inc.).

### Crystal Violet Staining

Crystal violet staining was used to analyze the effect of LCG-N25 on bacterial biofilm formation. The overnight culture of *S. mutans* was diluted to 1 × 10^5^ CFU/mL with BHIS. The bacterial suspension was mixed with the same volume of LCG-N25 and inoculated in a 96-well plate. LCG-N25’s final concentrations were set at 4, 2, 1, and 0.5 μg/mL based on the previous antimicrobial assays. The final concentration of DMSO was set at 0.004% in each experimental and control group. 200 μL of BHIS containing equal concentration of DMSO and bacterial suspension was used as a negative control. After incubated for 24 h at 37°C, the excess medium was removed, and the adherent biofilm was washed twice with sterile PBS. The biofilms in the wells were fixed by 200 μL of 4% (*w/v*) paraformaldehyde for 15 min. After the supernatant was eliminated, the plate was air-dried at room temperature, and 200 μL of 0.1% (*w/v*) crystal violet solution was added to each well and incubated for 5 min at room temperature. After eliminating the solution, the plate was washed twice with sterile PBS. Then 200 μL of 33% (*v/v*) acetic acid was added to dissolve the dye. The plates were shaken at room temperature for 30 min, and 100 μL of supernatant in each well was transferred to a new plate to record the absorbance at 575 nm.

### Colony Forming Units Counting

The effect of LCG-N25 on *S. mutans* biofilm formation was quantitatively assessed by counting CFU of live bacteria within biofilm. The overnight culture of *S. mutans* was diluted to 1 × 10^5^ CFU/mL in BHIS and the same volume of LCG-N25 were added to a 96-well plate. LCG-N25′s final concentrations were set at 4, 2, 1, and 0.5 μg/mL. The final concentration of DMSO was set at 0.004% in each experimental and control group. 200 μL of BHIS containing equal concentration of DMSO and bacterial suspension was used as a negative control. After anaerobic incubation at 37°C for 24 h, the supernatants from each well were decanted, and the plate was washed twice with PBS to remove the planktonic cells. The biofilm was re-suspended by 100 μL of PBS, and the bacterial suspension was diluted to 10^4^-fold to 10^6^-fold continuously. Then the suspensions were plated onto the BHI agar plates and incubated anaerobically at 37°C for 48 h to determine CFUs.

### Biofilm Metabolic Activity Assay

The metabolic activity of biofilm was assessed by Cell Counting Kit-8 (CCK-8) assay as described previously with minor modification (Sun et al., 2019).

For *S. mutans* biofilms formed in the presence of LCG-N25, a recovered culture of *S. mutans* was diluted to 1 × 10^5^ CFU/mL in BHIS and then added to a 24-well plate with an equal volume of LCG-N25 solution. The final concentrations of LCG-N25 were set from 4 to 1 μg/mL. The final concentration of DMSO was set at 0.004% in each experimental and control group. 200 μL of BHIS containing equal concentration of DMSO and bacterial suspension was used as a negative control. The plate was then incubated at 37°C for 24 h, then the medium was removed from the plate and the plate was rinsed twice with PBS. 100 μL of BHIS and 10 μl of CCK-8 reagent were then added to each well.

For the pre-established *S. mutans* biofilm, a recovered culture of *S. mutans* was diluted to 1 × 10^5^ CFU/mL in BHIS and then added to a 24-well plate. The plate was incubated at 37°C for 24 h, the medium was removed from the plate and the plate was then rinsed twice with PBS. BHIS with an equal volume of LCG-N25 solution were added to the plate. The final concentrations of LCG-N25 were set from 16 to 0.5 μg/mL. The final concentration of DMSO was set at 0.016% in each experimental and control group. After co-incubated at 37°C for 24 h, the medium was removed from the plate and the plate was rinsed twice with PBS. 100 μL of BHIS and 10 μl of CCK-8 reagent were then added to each well.

The plates were then incubated in dark at 37°C for 3 h, and the absorbance was measured at 450 nm using a spectrometer (Powerwave XS2, Bio-Tek, United States). CCK-8 assays were performed in triplicate.

### Quantification of Water-Insoluble EPS

The anthrone-sulfuric method was used for EPS detection in *S. mutans* as described previously (Ren et al., 2016). A recovered bacterial culture of *S. mutans* was diluted to 1 × 10^5^ CFU/mL in BHIS and then added to a 24-well plate with an equal volume of LCG-N25 solution. The final concentrations of LCG-N25 were set from 4 to 0.5 μg/mL. The final concentration of DMSO was set at 0.004% in each experimental and control group. 1,000 μL of BHIS containing equal concentration of DMSO and bacterial suspension was used as a negative control. The plate was then incubated at 37°C for 24 h, then the culture fluid was removed and the plate was washed twice with sterile PBS. Bacteria in each well was resuspended with sterile PBS. Each suspension was transferred to a sterile 1.5 mL centrifuge tube and centrifuged at 6,000 × *g* for 10 min at 4°C. The precipitate was then mixed with 1.0 M NaOH with agitation for 2 h at 37°C. The supernatant was collected and transferred into another centrifuge tube. Then 600 μL anthrone reagent (200 mg anthrone dissolved in 100 mL of sulfuric acid) was added into 200 μL of supernatant. The reaction mixture was heated in a water bath at 95°C and transferred to a new plate. The absorbance of each well was recorded at 625 nm.

### Scanning Electron Microscopy

The microstructure of bacterial biofilms was observed by Scanning Electron Microscopy (SEM). The recovered culture of *S. mutans* was diluted to 1 × 10^5^ CFU/mL in BHIS and was then added to a 12-well plate with an equal volume of LCG-N25 solution. Each well of the plate was added a glass coverslip for bacterial adhesion. The final concentration of LCG-N25 is set from 4 to 0.5 μg/mL. The final concentration of DMSO was set at 0.004% in each experimental and control group. 2 mL of BHIS containing equal concentration of DMSO and bacterial suspension was used as a negative control. The plate was then incubated at 37°C for 24 h. The supernatant was removed carefully and the plates were washed twice with sterile PBS. Then 2.5% (*v/v*) glutaraldehyde was added to the plates and kept at 4°C for 12 h to fix the biofilm. The biofilms were dehydrated by serial concentrations of ethanol (30, 40, 50, 60, 70, 80, 90, and 100%, *v/v*). Each concentration was treated for 15 min. After gold sputtering, the coverslips were observed under a SEM (Inspect F, FEI, Netherlands). Six fields of each coverslip were randomly selected for biofilm observation.

### Confocal Laser Scanning Microscopy

Confocal laser scanning microscopy (CLSM) was performed for EPS staining, dead/live imaging, and Fluorescence *in situ* hybridization (FISH). For EPS staining and dead/live imaging, the recovered culture of *S. mutans* was diluted to 1 × 10^5^ CFU/mL in BHIS; For FISH, the overnight cultures of *S. mutans*, *S. gordonii*, and *S. sanguinis* were simultaneously inoculated (inoculum ratio = 1: 1: 1) and diluted to 1 × 10^5^ CFU/mL in BHIS. The chemotaxis chamber μ-Slide with extremely low values of birefringence and autofluorescence (Zengel et al., 2011), was used for bacterial culture and confocal microscopy in this study. Bacterial suspensions and an equal volume of LCG-N25 solution were added to the μ-Slide (8 wells, 80826, Ibidi) at 37°C for 24 h. The final concentrations of LCG-N25 were set from 4 to 0.5 μg/mL. The final concentration of DMSO was set at 0.004% in each experimental and control group. BHIS containing equal concentration of DMSO and bacterial suspension was used as a negative control.

For extracellular polysaccharide (EPS) staining, the bacterial and the EPS were stained with SYTO 9 (Molecular Probes) and Alexa Fluor 647-labeled dextran conjugate (Molecular Probes). Alexa Fluor 647-labeled dextran conjugate was added to the wells at the beginning of biofilm formation, and SYTO9 was added on the 1-day-developed biofilms for 15 min of staining.

For dead/live imaging, the biofilms were stained with fluorescent LIVE/DEAD BacLight Bacterial Viability stain (Molecular Probes, Invitrogen) containing SYTO 9 and propidium iodide according to the manufacturer’s instructions for 15 min.

For FISH, the biofilms were fixed with 4% paraformaldehyde for 12 h and dried at 46°C. Then they were treated with 0.5 mL of buffer solution (50 mM EDTA, 100 mM Tris–HCl, pH 8.0) containing 30 mg/mL of lysozyme at 37°C for 20 min. The biofilms were rinsed and dehydrated with 50%, 80%, and 100% ethanol serially, and dried at 46°C for 10 min, followed by hybridization with species-specific probes (Supplementary Table 1). The biofilms were incubated with hybridization buffer [20 mM Tris–HCl (pH 8.0), 0.9 M NaCl, 20% formamide, 0.01% SDS] containing 2 nM of each specific probes at 46°C for 90 min in the dark and rinsed with preheated wash buffer [20 mM Tris–HCl (pH 8.0), 5 mM EDTA, 215 mM NaCl, 0.01% SDS] at 48°C for 15 min.

The samples were imaged with a DMIRE2 confocal laser scanning microscope (Leica, Wetzlar, Germany) equipped with a × 60 oil immersion lens. The microcopy images were analyzed using Image J COMSTAT software (NIH, United States) for quantification. Six fields were randomly selected to observe the biofilms.

### Bacterial Drug Resistance Assays

MIC measurements following repeated serial passages were performed to analyze whether *S. mutans* could develop drug resistance against LCG-N25 as described previously (Wang et al., 2017) with minor modification. CHX was used as a positive control. Briefly, the MIC values of LCG-N25 and CHX against *S. mutans* were measured as mentioned above. 100 μL portion of the bacterial suspensions in the sub-MIC well was taken and diluted by 10^4^-fold to 10^6^-fold in BHI. Then the suspensions were plated onto BHI agar plates and incubated anaerobically at 37°C for 48 h. Then the colonies were re-incubated with fresh BHI at 37°C with 5% CO_2_ for the next MIC test. All of the MIC tests were repeatedly performed for 20 passages. The potential incremental changes in MIC value through repeated passages were used to evaluate the development of drug resistance.

### *In vitro* Cytotoxicity/Viability Assay

Cell viability was evaluated in human oral keratinocytes (HOKs) and human gingival epithelial cells (HGEs) by using the CCK-8 (Dojindo, Kumamoto, Japan) assay. HOK and HGE cell lines were provided by the State Key Laboratory of Oral Diseases, Sichuan University. Cells were plated in 96-well plates at a density of 1 × 10^5^ cells/well with the Dulbecco’s modified Eagle’s medium (DMEM) supplemented with 10% fetal bovine serum and 1% antibiotic-antimycotic in 5% CO_2_ at 37°C for 24 h. Cells were cultured with media containing LCG-N25 and NAP (final concentrations from 0.25 to 128μg/mL) for 5 min/24 h. The final concentration of DMSO was set at 0.128% in each experimental and control group. CHX was used as a positive control. Fresh medium was used as blank control. Then, the cells were washed with PBS twice. 100 μL of medium and 10 μL of CCK-8 were then added to each wall. After incubation in the CO_2_ incubator for 2 h, absorbance was measured at the wavelength of 450 nm. The cell viability was calculated according to the following formula (%): = (*A*_450__ nm_ of test group − *A*_450__ nm_ of blank control)/(*A*_450__ nm_ of negative control − *A*_450__ nm_ of blank control) × 100%.

### Statistical Analysis

All the experiments were performed in triplicate. Statistical analyses were performed using SPSS 16.0 (SPSS, Inc., Chicago, IL, United States). The statistical results were expressed as mean ± standard deviation (SD). After verifying the equal variance assumptions of the data, the results were analyzed by one-way analysis of variance (ANOVA), followed by Tukey multiple comparison tests. Quantitative analyses of microbial composition in the fluorescence-labeled three-species biofilm were analyzed with chi-squared test. Differences were considered significant at a *P*-value < 0.05.

## Results

### Chemical Synthesis of Compound LCG-N25

The synthesis of the target compound LCG-N25 was carried out following the procedures shown in Figure 1. The intermediate 2 was prepared from the starting material, 2,3-dichloronaphthalene-1,4-dione, according to the previously reported procedure (Santos et al., 2010). LCG-N25 was obtained by an ammonolysis of intermediate 2.

### LCG-N25 Shows Good Antimicrobial Activity Against Oral Streptococci

LCG-N25 was bactericidal against *S. mutans, S. gordonii*, and *S. sanguinis* planktonic cells with MICs ranging from 0.125 to 0.5 μg/mL and MBCs ranging from 8 to 15.6 μg/mL (Table 1).

**TABLE 1:** MIC and MBCs of LCG-N25, NAP, and CHX against *S. mutans*, *S. gordonii*, and *S. sanguinis*.

Bacterial species	MIC^a^ (μg/ml)			MBC^b^ (μg/ml)		
	LCG-N25	CHX	NAP	LCG-N25	CHX	NAP
*S. mutans*	0.5	1.56	3.91	15.6	7.81	15.63
*S. gordonii*	0.125	3.91	0.49	8	7.81	0.98
*S. sanguinis*	0.125	0.49	0.49	15.6	3.91	15.63

*^a^MIC, minimal inhibitory concentrations. ^b^MBC, minimal bactericidal concentration.*

**FIGURE 2:**
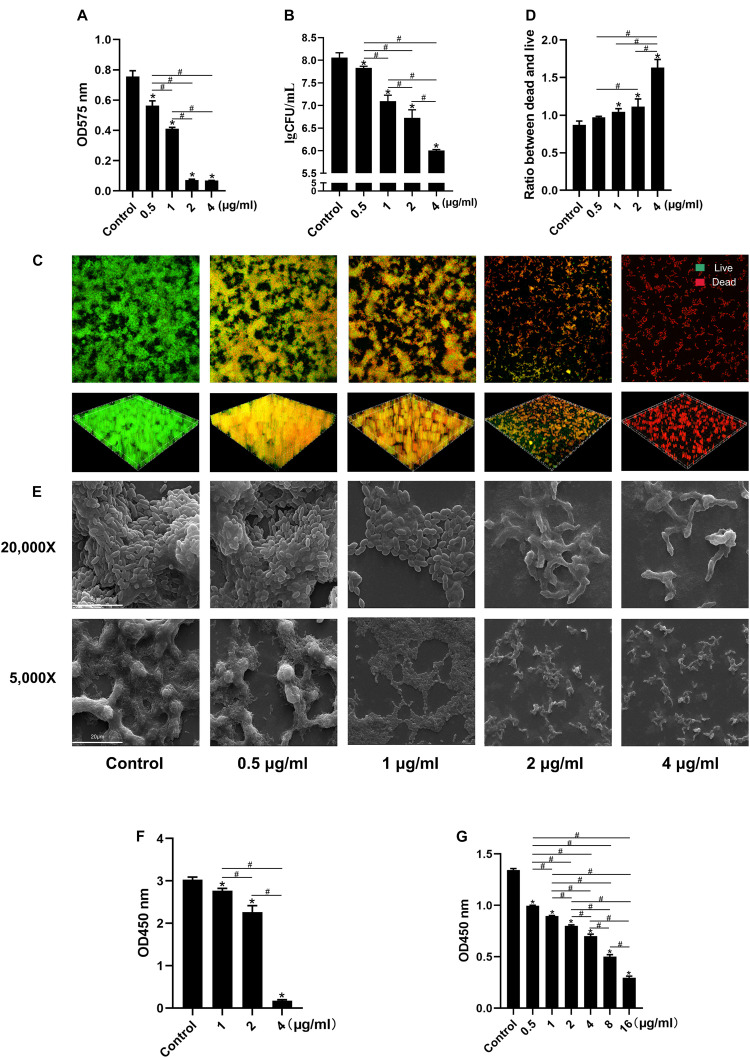
Effect of LCG-N25 on *S. mutans* biofilm. **(A)** Biomass of biofilm formed by *S. mutans* in the presence of LCG-N25 as determined by crystal violet staining. **(B)** Viable bacterial counts in the *S. mutans* biofilm formed in the presence of LCG-N25. **(C)** Representative images of dead/live bacteria within the *S. mutans* biofilm formed in the presence of LCG-N25. **(D)** Quantitative analyses of dead/live bacterial in the treated biofilm. **(E)** Representative SEM images of *S. mutans* biofilm formed in the presence of LCG-N25. Observation the microstructure of bacterial biofilms after treatment of LCG-N25. **(F)** Metabolic activity of *S. mutans* biofilms formed in the presence of LCG-N25. **(G)** Metabolic activity of pre-established *S. mutans* biofilm after treatment with LCG-N25. Data are presented as means ± standard deviations from three independent experiments. Six fields were randomly selected to observe the biofilms in each sample. *Statistically significant differences as compared with control (*P* < 0.05). ^#^Statistically significant differences between treatment groups (*P* < 0.05).

LCG-N25 also showed good antimicrobial activity against *S. mutans* biofilm. The result of crystal violet staining showed that LCG-N25 treatment decreased the biomass of *S. mutans* biofilm (Figure 2A). Consistently, LCG-N25 treatment dose-dependently reduced live bacteria within biofilms as reflected by both CFU counting and live/dead fluorescent staining (Figures 2B–D). Images obtained by SEM further confirmed that LCG-N25 significantly reduced the total biomass in the biofilm and disrupted its structure (Figure 2E). LCG-N25 also showed good inhibitory effects on the metabolic activity of biofilm. The metabolic activities of *S. mutans* biofilms formed in the presence of varied concentrations of LCG-N25 were significantly inhibited (Figure 2F). Moreover, LCG-N25 dose-dependently reduced the metabolic activities of pre-established biofilms, as 16 μg/ml of LCG-N25 nearly caused an 80% reduction in the metabolic activity of *S. mutans* biofilms (Figure 2G).

**FIGURE 3:**
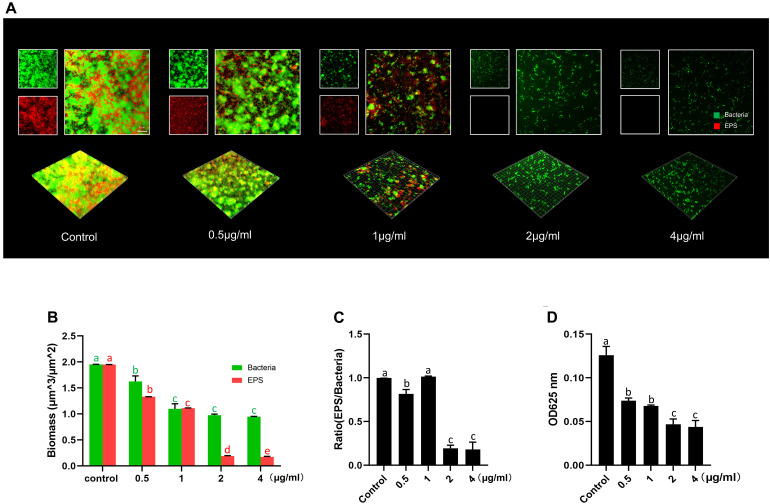
The inhibitory effect of LCG-N25 on the EPS synthesis of *S. mutans*. **(A)** Representative images of *S. mutans* biofilm treated with LCG-N25, imaged by CLSM. **(B)** Quantification of the amounts of EPS and bacteria within *S. mutans* biofilms. **(C)** The ratio of EPS/bacteria within the biofilms. **(D)** Quantitative determination of water-insoluble EPS in *S. mutans* biofilm using anthrone method. The absorbance was recorded at 625 nm. Data are presented as means ± standard deviations from three independent experiments. Groups identified by distinct lowercase letters are statistically different (*P* < 0.05).

Further analyses of the EPS of *S. mutans* biofilm by CLSM showed that LCG-N25 treatment reduced both bacteria and EPS, altered the EPS/bacterium ratio of the biofilms in a dose-dependent manner as compared to the non-treated control (Figures 3A–C). Consistently, anthrone assay showed that LCG-N25 treatment significantly reduced the EPS production of *S. mutans* (Figure 3D).

### LCG-N25 Altered the Microbial Composition of the Multi-Species Biofilm

**FIGURE 4:**
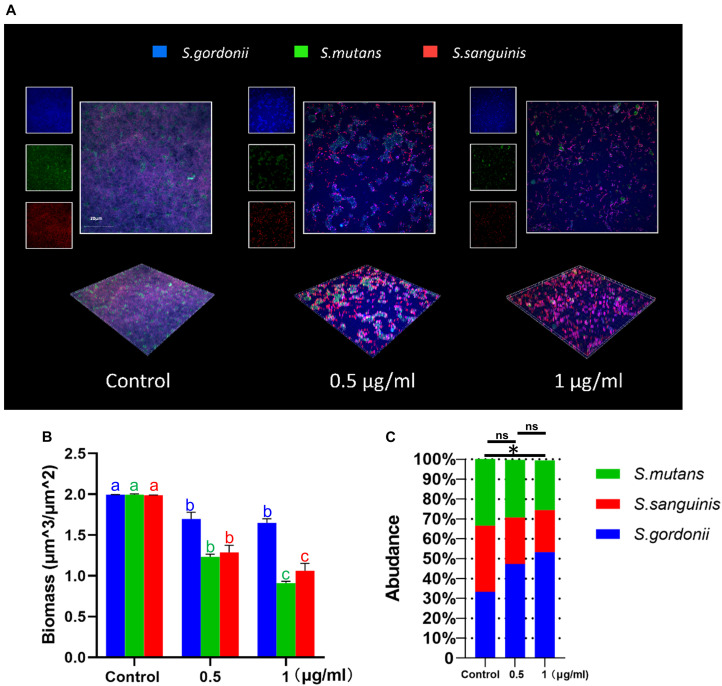
The effect of LCG-N25 on the microbial composition of multi-species streptococcal biofilms. **(A)** Representative FISH images of multi-species biofilms in the presence of LCG-N25. **(B)** Quantification of the amounts of *S. mutans*, *S. gordonii*, and *S. sanguinis* in multi-species biofilms. **(C)** The ratios of *S. mutans*, *S. gordonii*, and *S. sanguinis* in multi-species biofilms were quantified by FISH. Data are presented as means ± standard deviations from three independent experiments. Six fields were randomly selected to observe the biofilms in each sample. Groups identified by distinct lowercase letters are statistically different (*P* < 0.05). ^∗^Statistically significant differences compared to the control (*P* < 0.05); ns, not significant.

The effects of LCG-N25 on the microbial composition of multispecies biofilms were investigated by species-specific FISH. LCG-N25 treatment reduced the biomass of *S. mutans, S. sanguinis*, and *S. gordonii* in multi-species biofilm. Moreover, the relative abundance of *S. mutans* in the three-species biofilms was considerably reduced after LCG-N25 treatment as compared to the non-treated control. However, the relative abundances of *S. gordonii* in the three-species biofilms increased after LCG-N25 treatment (Figures 4A–C), indicating a selectivity of LCG-N25 against *S. mutans* within the mixed biofilm.

### LCG-N25 Induces No Drug Resistance in *Streptococcus mutans*

**FIGURE 5:**
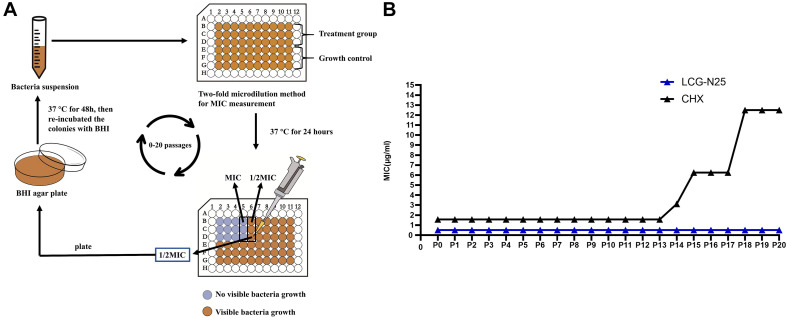
**(A)** Schematic illustration of broth microdilution method used for the resistance assay. **(B)** MIC values of LCG-N25 and CHX against *S. mutans* from Passage 0 to 20 (P0–P20). Data are presented as means ± standard deviations from three independent experiments.

To evaluate whether LCG-N25 could induce drug resistance against *S. mutans*, the MIC values of LCG-N25 and CHX from passages 0 to 20 were measured Figure 5A and shown in Figure 5B. For LCG-N25, the MIC values for *S. mutans* stabilized at 0.5μg/ml from P0 to P20, indicating LCG-N25 didn’t induce drug resistance against *S. mutans* within 20 passages. As for CHX, the MIC values for *S. mutans* increased from 1.56 to 12.5 μg/mL within 20 passages. These results indicated that LCG-N25 was less likely to induce drug resistance in *S. mutans* as compared to CHX.

### LCG-N25 Exhibits Low Cytotoxicity to Common Human Oral Cells

**FIGURE 6:**
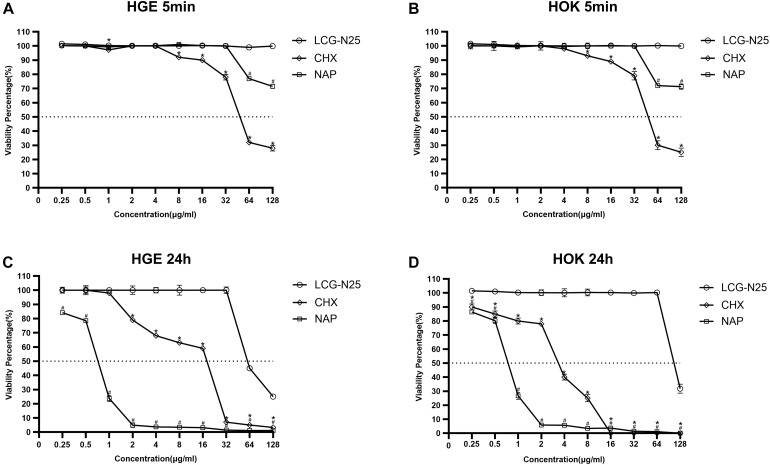
Cytotoxicity of LCG-N25 on human oral keratinocytes and human gingival epithelial cells. The cell viability was determined by CCK-8 assay after cultured with/without LCG-N25/CHX/NAP for either 5 min **(A,B)** or 24 h **(C,D)**. Data are presented as means ± standard deviations from three independent experiments. *Statistically significant differences between CHX and LCG-N25 (*P* < 0.05). ^#^Statistically significant differences between NAP and LCG-N25 (*P* < 0.05).

The cytotoxicity of LCG-N25 against HOKs and HGEs was evaluated by CCK-8 assay. As shown in Figures 6A,B, LCG-N25 exhibited low cytotoxicity against HOK and HGE cells in an exposure duration of 5 min [50% inhibitory concentration (IC50), >128 μg/ml]. Besides, when the treated concentration was higher than 8 μg/ml, LCG-N25 showed lower cytotoxicity than CHX; when the treated concentration was higher than 32 μg/ml, LCG-N25 showed lower cytotoxicity than NAP. In addition, exposure of LCG-N25 for 24 h didn’t show significant inhibition of cell proliferation either [(IC50), 128 μg/ml for HOK; 64 μg/ml for HGE]. However, CHX and NAP showed considerable cytotoxicity in an exposure duration of 24 h [CHX: (IC50), 4 μg/ml for HOK; 32 μg/ml for HGE] [NAP: (IC50), 1 μg/ml for HOK; 1 μg/ml for HGE] (Figures 6C,D).

## Discussion

Dental plaque biofilm plays a critical role in the development of dental caries, and plaque biofilm control is effective in dental caries prevention and management. Plaque control measures such as tooth brushing and flossing are considered effective in caries prevention (Axelsson et al., 1976). However, it is difficult to eliminate cariogenic bacteria from pits, fissures, and approximal surfaces of teeth merely by mechanical methods (Agarwal and Nagesh, 2011). Therefore, combinatory use of antimicrobials is necessary for effective caries control, particularly for the high-risk population. CHX has been used in dentistry for nearly 40 years and remains the gold standard compared with other antimicrobial agents (Jones, 1997). However, CHX could induce microbial resistance (Wang et al., 2017), and cause tooth discoloration and taste confusions (Cabral and Fernandes, 2007), necessitating the development of alternatives to CHX for the better control of dental caries. In the present study, we redesigned and synthesized a novel small molecule, based on a lead compound, showing potential in plaque biofilm control. This novel small molecule, LCG-N25, exhibited potent antimicrobial activity against oral streptococcal biofilms, low cytotoxicity against human oral cells, and induced no antimicrobial resistance in *S. mutans*, representing a promising antimicrobial agent that can be used in the clinical management of dental caries.

Small molecules have shown the potential as antimicrobial agents due to their good antibacterial activity, good stability, and low toxicity (Worthington et al., 2012; Nizalapur et al., 2016). Structural modification and optimization based on the promising lead compounds are useful approaches in drug discovery and development (Yang et al., 2019; Shi et al., 2020). Previous studies have demonstrated that structural modification of lead compounds can enhance its inhibitory activity against microorganisms, as well as improve its cell potency and pharmacokinetic profiles but reduce its toxicity to human cells (Yang et al., 2019; Shi et al., 2020; Zhang et al., 2020). We previously screened a library of bioactive molecules against *S. mutans* and identified a natural compound NAP showing good antimicrobial activity against oral streptococci (Kuang et al., 2020). However, as an antitumor treatment currently in phase III clinical trials (Sonbol et al., 2019), the mild cytotoxicity of NAP on oral cells may limit its repurposed used in the control of dental caries. The current study redesigned a novel small molecule LCG-N25 using NAP as a lead compound, expecting to enhance its antimicrobial activity while reducing cytotoxicity. Our data showed that LCG-N25 exhibited significantly lower cytotoxicity on HOKs and HGEs as compared to CHX and NAP. In addition, the antimicrobial activities of LCG-N25 against oral streptococci were significantly enhanced after structural modifications, with almost an eight-fold decrease of MIC against *S. mutans* as compared to that of NAP (3.91 μg/mL as reported previously) (Kuang et al., 2020), and a 3-fold decrease in MIC as compared to that of CHX. All these data indicate that LCG-N25 is more suitable for the control of oral biofilms as compared to its lead compound NAP. Of note, in the past decades, natural products such as propolis, magnolia bark, cinnamaldehyde have also shown good potential in caries control with good inhibitory effects on the microbial growth and virulence of oral streptococci (Jeon et al., 2011; Cheng et al., 2015). However, the solubility and chemical stability challenge their clinical translation. In addition, MICs of natural compounds against oral streptococci are usually higher than 16 μg/mL, which are much higher than antimicrobial small molecules (e.g., the MIC of LCG-N25 against *S. mutans* was 0.5 μg/mL in the current study), further suggesting the good translational potential of LCG-N25 in the control of dental caries.

The formation of oral biofilm is a multi-step process, including bacterial adhesion, EPS production and biomass accumulation (Flemming et al., 2016). EPS production mediates microbial adherence to tooth surface and cell-to-cell adhesion directly, while forming a polymeric matrix which can enhance mechanical stability of biofilms and help oral pathogens create pathogenic potentials (Bowen et al., 2018). Thus, EPS is recognized as crucial virulence factor associated with dental caries (Bowen and Koo, 2011), and it is usually employed as the potential target for the development of novel therapeutics to disrupt oral biofilm and control caries (Balakrishnan et al., 2000; Koo et al., 2013). In the present study, we also found that LCG-N25 could significantly inhibit the EPS production of *S. mutans*, which further provides evidence to its clinical application as a dental plaque control agent.

According to the ecological plaque hypothesis, dental caries is more associated with microbial disequilibrium rather than the virulence properties of single species (Kuramitsu and Wang, 2006; Li et al., 2010; Samaranayake and Matsubara, 2017). One of the major concerns on the topical use of broad-spectrum antimicrobials as anticaries measures is the possible cause of oral microbial dysbiosis (Chen et al., 2020). CHX as a broad-spectrum antimicrobial agent can effectively inhibit most of oral microbes but with poor selectivity, which might impose adverse effects on the oral microbial ecology after long-term use (Tokajuk et al., 2017; Sakaue et al., 2018). A multispecies biofilm model consisting of *S. mutans*, *S. sanguinis*, and *S. gordonii* was reported previously that could be used to evaluate the ecological impact of the tested compounds on oral biofilms (Zhang et al., 2019; Yu et al., 2020). In the current study, although *S. mutans* was less sensitive to LCG-N25 (MIC = 0.5 μg/mL) relative to the other 2 streptococci (MICs = 0.125 μg/mL) in the planktonic culture, data obtained from FISH showed that treatment with LCG-N25 led to a decreased proportion of *S. mutans* and an elevation of other commensal streptococci within mixed biofilms. These data suggest that LCG-N25 may not impose significant impact on oral ecosystem when used as a plaque control agent. Although the underlying mechanisms for this selectivity in the mixed biofilm are not investigated in the current study, we speculate that the inter-species interactions as well as the barriers effect of extracellular matrix of the biofilms may benefit the better selectivity of LCG-N25 against *S. mutans* within the mixed biofilm. Further studies are warranted to investigate the underlying molecular mechanisms.

Bacterial drug resistance is one of the greatest threats to human health (Kumar and Balbach, 2017). The widespread of antimicrobial resistance could limit clinical treatment options for microbial infections (Hegstad et al., 2010). Long-term use of CHX could cause the development of microbial resistance in microbes, including *Enterococcus faecalis*, *Klebsiella pneumoniae*, *Staphylococcus aureusa*, and *Pseudomonas aeruginosa* (Wang et al., 2017). Consistently, our study also found that repeated exposure of *S. mutans* to sublethal level of CHX could develop conspicuous resistance after 15 passages. However, the MIC values for LCG-N25 against *S. mutans* were stable at 0.5 μg/ml from P0 to P20, suggesting that this novel compound would not induce drug resistance in *S. mutans* and may have good potential as a daily-use antimicrobial mouth rinse for the plaque biofilm control.

In conclusion, this study synthesized a novel small molecule LCG-N25 based on a previously identified natural lead compound. LCG-N25 exhibited a good antibacterial activity, low-cytotoxicity, and did not induce drug resistance of cariogenic *S. mutans*, representing a promising adjuvant to the management of dental caries.

## Data Availability Statement

The datasets presented in this study can be found in online repositories. The names of the repository/repositories and accession number(s) can be found in the article/Supplementary Material.

## Author Contributions

YL and XX conceived and designed the experiment. XL, CL, JZ, and LW performed the experiments. XL, YS, LC, and QJ performed the statistical analysis. XL wrote the first draft of the manuscript. CL, YL, and XX helped to revise the manuscript. All authors have read and agreed to the published version of the manuscript.

## Conflict of Interest

The authors declare that the research was conducted in the absence of any commercial or financial relationships that could be construed as a potential conflict of interest.
