# Supplementary material for: A Novel Small Molecule, LCG-N25, Inhibits Oral Streptococcal Biofilm
Source: Front Microbiol. 2021 Mar 29;12:654692. doi: 10.3389/fmicb.2021.654692 (PMC8044806; doi:10.3389/fmicb.2021.654692)
Supplement: Supplementary file 1 [file Table_1.docx]

Supplementary Material

**CONTENTS:**

**FIGURE S1**: ^1^H NMR of intermediate 2

**FIGURE S2**: ^1^H NMR of LCG-N25

**FIGURE S3**:^13^C NMR of LCG-N25

**FIGURE S4**: HRMS of LCG-N25

**FIGURE S5**: The ratios of *S. mutans*, *S. gordonii*, and *S. sanguinis* in multi-species biofilms

**Table S1:** Probes used for fluorescent in situ hybridization

**
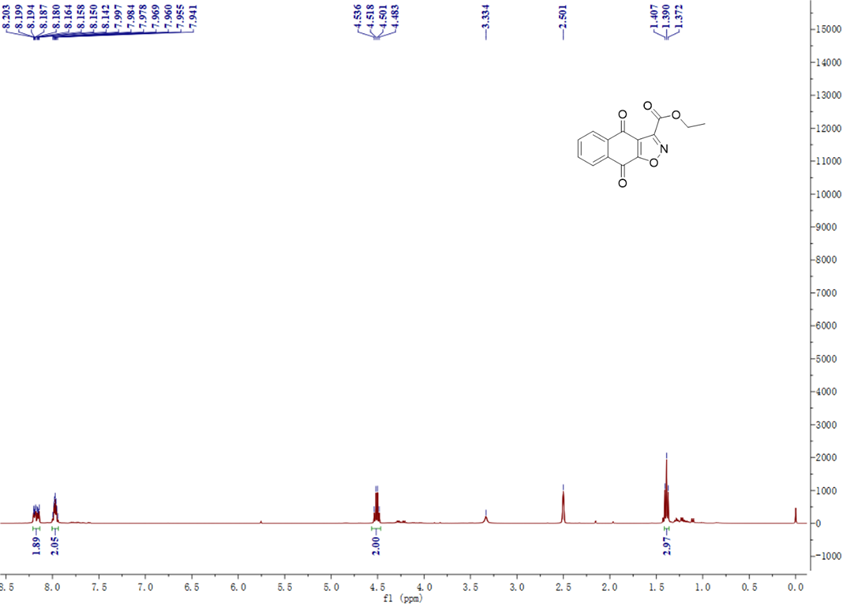
**

**Supplementary Figure 1.** ^1^H NMR of intermediate 2. (400 MHz, DMSO) δ 8.24 – 8.11 (m, 2H), 8.02 – 7.91 (m, 2H), 4.51 (q, J = 7.1 Hz, 2H), 1.39 (t, J = 7.1 Hz, 3H).


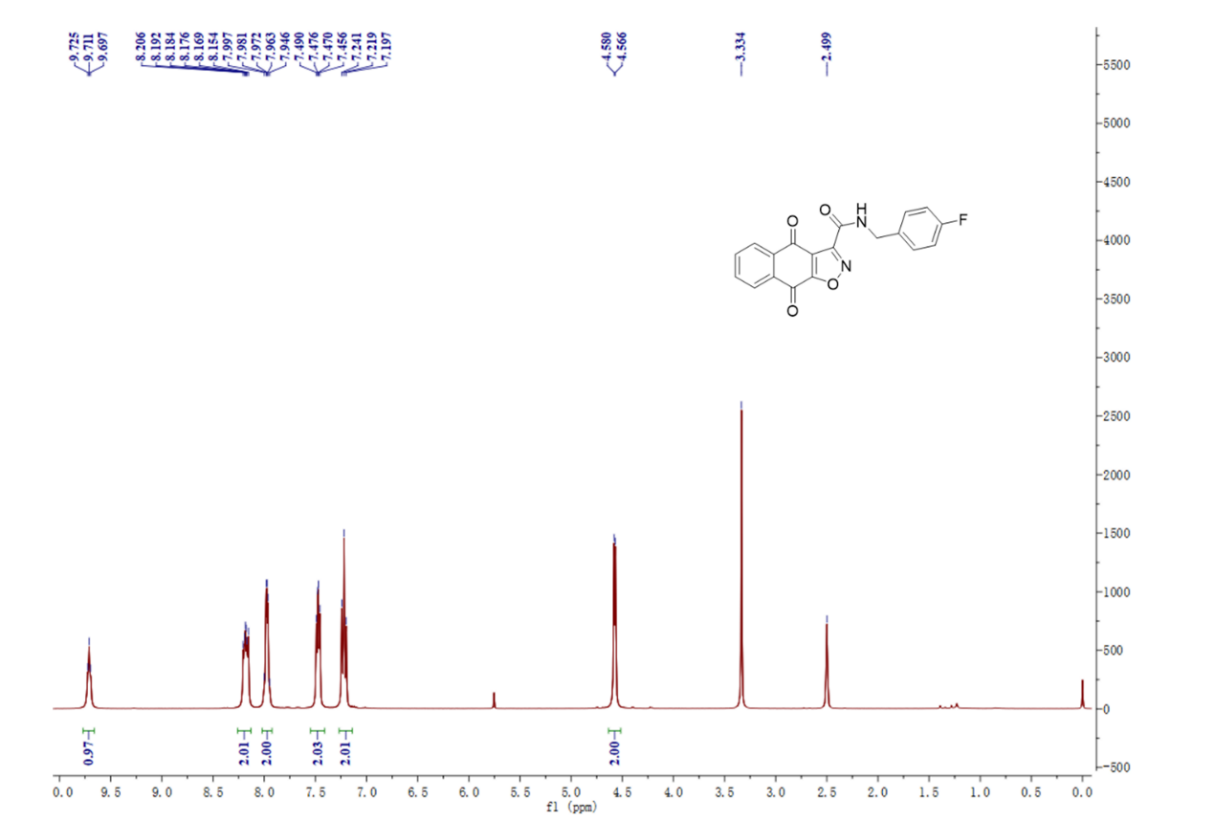


**Supplementary Figure 2.** ^1^H NMR of LCG-N25. (400 MHz, DMSO) δ 9.71 (t, J = 5.7 Hz, 1H), 8.27 – 8.11 (m, 2H), 7.97 (m, 2H), 7.47 (dd, J = 8.2, 5.7 Hz, 2H), 7.22 (t, J = 8.8 Hz, 2H), 4.57 (d, J = 5.7 Hz, 2H).


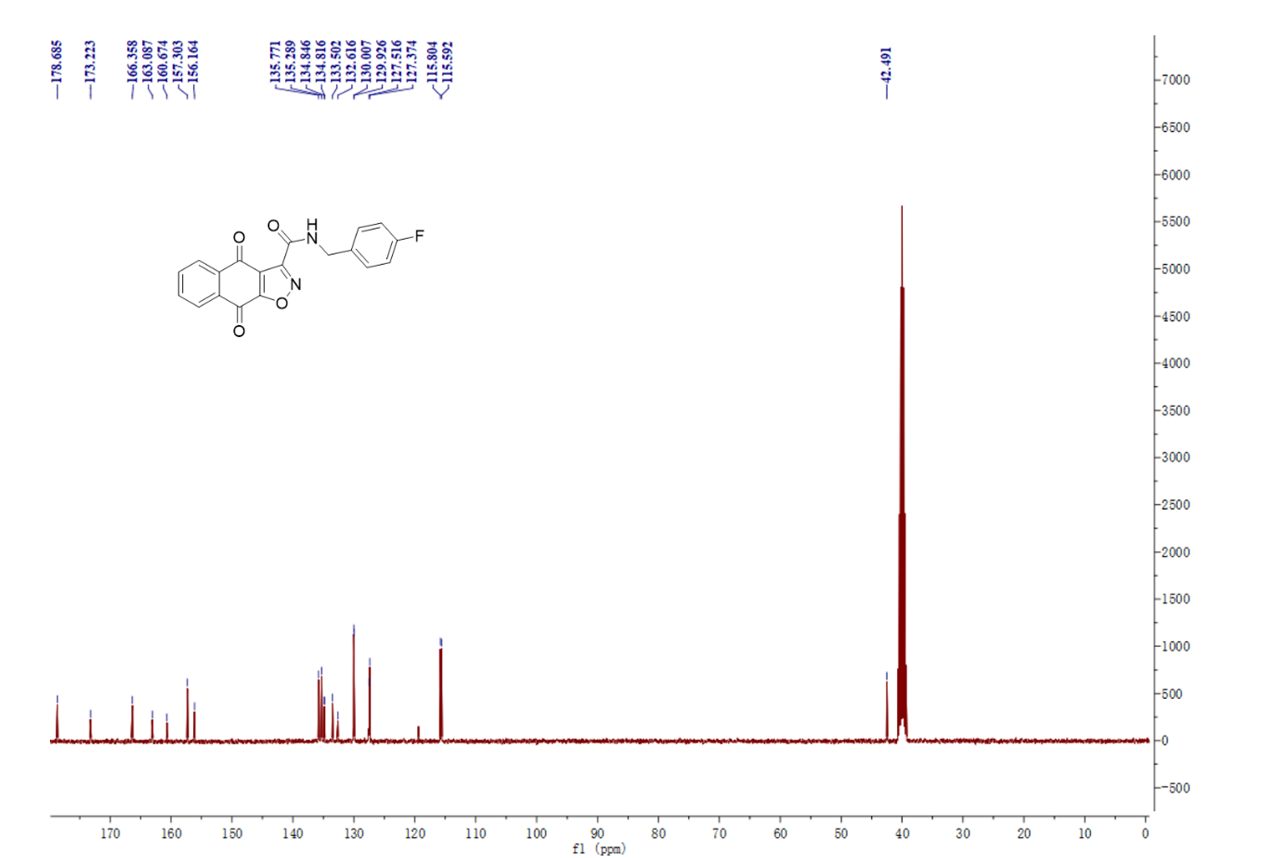


**Supplementary Figure 3.** ^13^C NMR of LCG-N25. (100 MHz, DMSO) δ 178.69, 173.22, 166.36, 163.09, 160.67, 157.30, 156.16, 135.77, 135.29, 134.85, 134.82, 133.50, 132.62, 130.01, 129.93, 127.52, 127.37, 115.80, 115.59, 42.49.


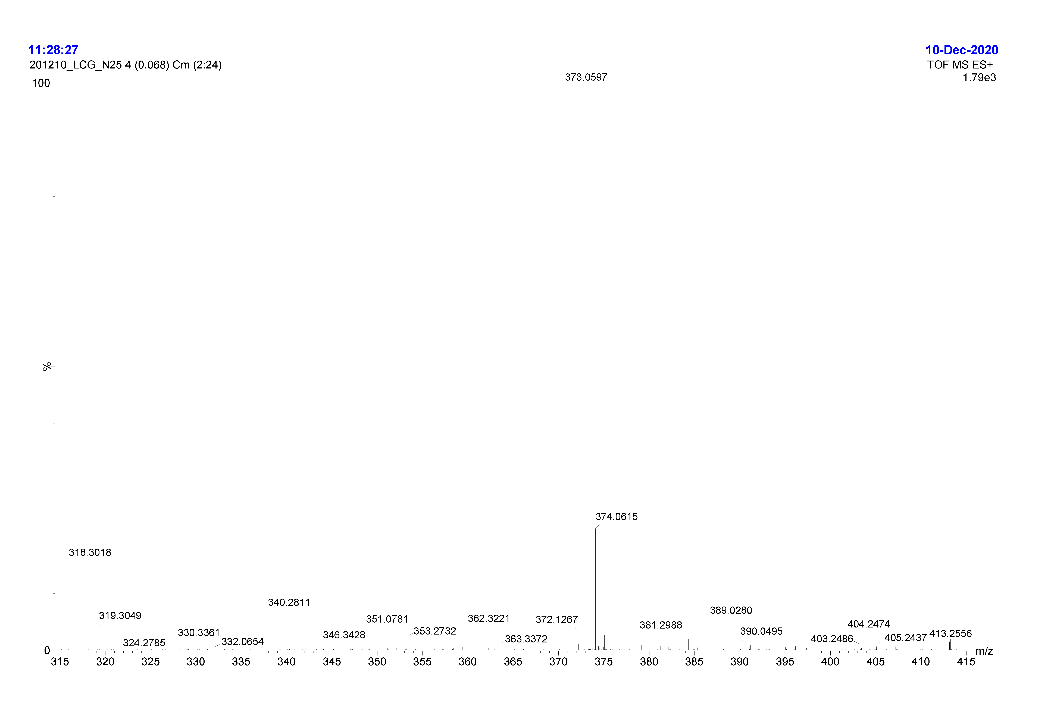


**Supplementary Figure 4.** HRMS (Q-TOF) of LCG-N25. Calculated for C19H11FN2O4 373.0601 [M+Na]+, found 373.0597.


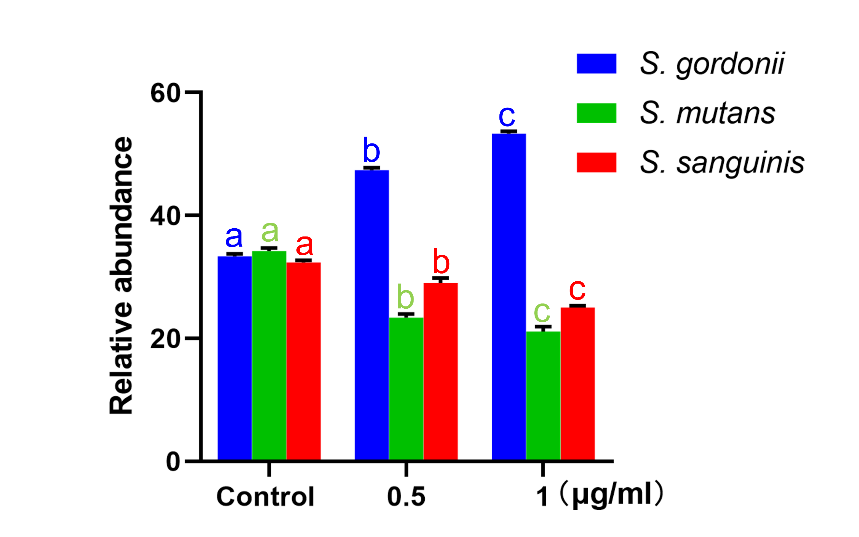


**Supplementary Figure 5.** The ratios of *S. mutans*, *S. gordonii*, and *S. sanguinis* in multi-species biofilms were quantified by FISH. Data are presented as means ± standard deviations from three independent experiments. Six fields were randomly selected to observe the biofilms in each sample. Groups identified by distinct lowercase letters are statistically different (P <0.05).

**Supplementary Table 1.** Probes used in fluorescent in situ hybridization

| **Probes** | **Sequenes (5′-3′)** |
| --- | --- |
| ***S. mutans*** | **ACTCCAGACTTTCCTGAC** |
| ***S. sanguinis*** | **GCATACTATGGTTAAGCCACAGCC** |
| ***S. gordonii*** | **ACTGTGCGTTCTACTTGC** |
